# The Time Has Come to Understand the Mechanisms by Which Comorbidities Contribute to COVID-19 Severity

**DOI:** 10.3390/jpm12020123

**Published:** 2022-01-18

**Authors:** Bruno Mégarbane

**Affiliations:** Department of Medical and Toxicological Critical Care, Lariboisière Hospital, INSERM UMRS-1144, Paris-University, 75010 Paris, France; bruno.megarbane@lrb.aphp.fr; Tel.: +33-149-958-442

A new coronavirus named severe acute respiratory syndrome coronavirus-2 (SARS-CoV-2) has been responsible for a worldwide pandemic for two years, resulting in almost 280 million infections and 5.4 million deaths at the end of 2021. As soon as the pandemic emerged, bedside observations identified patients at higher risk of developing severe coronavirus disease-2019 (COVID-19)-attributed pneumonia and fatality [1,2,3,4]. Age was the most important factor associated with death. Comorbidities including diabetes; obesity; past hypertension; cancer; and chronic kidney, cardiovascular, and respiratory diseases were associated with an increased risk of developing severe respiratory symptoms, leading to hospitalization or even intensive care unit admission. Refractory acute respiratory injury despite the use of high-flux oxygen, mechanical ventilation, and even extracorporeal membrane oxygenation as well as bacterial and mycological superinfections, thromboembolic complications, and multiorgan failure represented the causes of death in the most severe cases. Various anti-COVID-19 therapies, including repurposed antiviral, immunomodulatory, and anticoagulant drugs, were extensively used in addition to supportive care but remained insufficiently effective [5].

The question of understanding how comorbidities may contribute to COVID-19 severity is still unanswered. Whether underlying comorbidities increase the probability of contamination by facilitating SARS-CoV-2 entry or replication in the targeted cells, thus enhancing the viral load; whether they alter the host’s immune response to the viral infection by precipitating the cytokine storm; or whether they just enhance the burden of the disease in frailer patients is worthy investigating. Understanding the possibly involved molecular mechanisms can help design highly effective targeted anti-COVID-19 therapies for the future.

Gene polymorphism was first hypothesized to explain a predisposition to developing severe COVID-19 presentations. The observed increased vulnerability in particular patient subgroups led to search using genome-wide association (GWAS) for gene signals involved in key host antiviral defense mechanisms and/or mediators of inflammatory organ damage [6]. Putative loss-of-function variants of X-chromosomal Toll-like receptor (TLR)7 associated with impaired type-I and II interferons responses were identified in four critically ill SARS-CoV-2 infected young males, providing insight into COVID-19 pathogenesis and underlying the essential protective role of type-I interferon immunity against SARS-CoV-2 [7]. Later, X-linked recessive TLR7 deficiency was demonstrated to be a highly penetrant genetic etiology of critical COVID-19 pneumonia with a prevalence of ~1.8% in male patients below the age of 60 years [8].

The implication of other gene candidates facilitating viral replication, altering the immune response, and/or explaining interindividual differences in innate and adaptative immune responses was investigated. Inborn errors of TLR3- and IRF7-dependent type-I interferon immunity were shown to underlie life-threatening COVID-19 pneumonia [9]. Based on international cohorts, B cell autoimmune phenocopy of inborn errors of type-I interferon immunity was shown to account for life-threatening COVID-19 pneumonia in at least 2.6% of women and 12.5% of men [10]. Autoantibodies neutralizing type-I interferons, predating SARS-CoV-2 infection and sharply increasing in prevalence after the age of 70 years, were found to account for ~20% of critically ill COVID-19 patients over 80 and total fatalities [11]. Additionally, a strategy for identifying, recruiting, and genetically analyzing individuals who are naturally resistant to SARS-CoV-2 infection was developed based on a global network of researchers [12]. A meta-analysis including 50,000 patients from 46 studies confirmed the protective effect, although weak (odds ratio of ~0.90) of the O allele among the ABO blood groups, which may serve as co-receptors for SARS-CoV-2 [13]. A variant located close to the angiotensin-converting enzyme-2 (ACE2), the key cell receptor of SARS-CoV2, was identified more recently using GWAS as able to confer protection, possibly by decreasing ACE2 expression on SARS-CoV-2-targeted cells [14]. Using genome-wide CRISPR knockout screen for SARS-CoV-2 infection, an endoplasmic reticulum transmembrane protein called Transmembrane Protein 41B (TMEM41B) was recognized as essential for permissive infection, with a potential relationship between its level of expression and COVID-19 severity [15].

However, gene polymorphism could not explain all individual vulnerabilities. Alterations in relation to comorbidities of the expression of genes involved in type-I interferon signaling and in viral-replication pathways have been suggested. In addition to ACE2, heparan sulfate proteoglycans were found to interact with the viral spike protein, explaining why variations in their expression could result in increased susceptibility to COVID-19 [16]. Since SARS-CoV-2 entry requires sequential cleavage of the spike protein, synthesized as an inactive precursor, at the S1/S2 and the S2ʹ cleavage sites to mediate membrane fusion, proteases involved in these processes were suggested to influence individual susceptibility to COVID-19 if their baseline activity could vary according to comorbidities. Cell surface proteolytic enzymes, such as the transmembrane serine protease isoform 2 (TMPRSS2) and the human airway trypsin-like protease (HAT or TMPRSS11D), cleave SARS-CoV-2 spike protein at the cell surface during attachment [17,18]. Other transmembrane serine proteases (TTSPs) and cathepsins (primarily cathepsin L), located in the endolysosomes, further contribute to priming the spike protein and degrading the extracellular matrix to allow SARS-CoV-2 to enter the host cells. In addition, the identification of a unique insertion of four amino acids (PRRA) at the S1/S2 boundary in the sequence of SARS-CoV-2 spike protein suggested that furin or furin-like proteases could be involved when virions are made in the host cells and influence the level of viral replication and thus the severity of the resulting disease.

Both the renin-angiotensin-aldosterone and the kinin–kallikrein systems are implicated in the cytokine storm that may result in COVID-19 patients [19,20]. SARS-CoV-2-induced ACE2 inhibition leads to the augmentation of bradykinin 1-receptor effects, as ACE2 inactivates des-Arg9-bradykinin, a bradykinin metabolite. SARS-CoV-2 decreases the effects of bradykinin 2-receptor as it affects bradykinin synthesis by inhibiting cathepsin L, present at the site of infection and involved in bradykinin production. Dysregulation of the renin-angiotensin-aldosterone balance and overactivation of angiotensin II type-I receptor axis could result in vasoconstriction and induction of the pro-fibrotic, apoptotic, and inflammatory signalizations in SARS-CoV-2-targeted tissues [21].

In work published in the journal and using various SARS-CoV-2-targeted organ tissues obtained from 1968 patients with common comorbidities known to enhance the risk of COVID-19 severity, Breidenbach and colleagues studied the expression of various genes that might facilitate SARS-CoV-2 entry [22]. Gene expression was mapped using single-cell RNA sequencing in whole-tissue homogenate and compared to international databases. Interestingly, the tissue site of the greatest gene expression varied according to the involved comorbidity, suggesting complex and non-uniform underlying mechanisms supporting patient vulnerability. Accordingly, ACE2 expression was the greatest in the pulmonary tissues of cancer patients, in the kidney tissues of obese patients, in the cardiac tissues of heart failure diabetic patients, and in the peripheral blood mononuclear cells of coronary disease patients. A significant increase in the expression of TTSPs, including TMPRSS2 and HAT, was found across the SARS-CoV-2-targeted organ systems in patients with hypertension, cancer, and history of smoking in comparison to healthy individuals. Together with molecular biology studies investigating gene induction, inhibition, or knockout, Breidenbach’s data supported the contribution of SARS-CoV-2 entry-related gene expression patterns to the outcome of COVID-19 patients. However, as acknowledged by these researchers, their findings were limited by their whole-tissue-homogenate-based approach, which included the vascular component of each tissue type, since SARS-CoV-2-mediated endothelial alterations are a well-established non-organ-specific injury related at least in part to modifications in bradykinin receptor expression and activity [23]. Therefore, these preliminary findings still have to be interpreted with caution before correlating the observed tissue-specific gene expression patterns with the involved comorbidity.

To conclude, there is still a long way to go to improve our understanding of the vulnerability of patients with comorbidities to develop severe patterns of COVID-19. All the work undertaken will also be very useful to shed light on the pathophysiology of SARS-CoV-2 infection and to develop specific curative and preventive therapies for the coming years in order to protect at-risk populations and reduce the resulting morbidity and mortality.

## Data Availability

No new data were created or analyzed in this study. Data sharing is not applicable to this article.
